# The potential role of HPV oncoproteins in the PD-L1/PD-1 pathway in cervical cancer: new perspectives on cervical cancer immunotherapy

**DOI:** 10.3389/fonc.2024.1488730

**Published:** 2024-12-13

**Authors:** Jin Li, Yuhong Ma, Qixuemeng Wu, Pengbin Ping, Juan Li, Xiaoying Xu

**Affiliations:** Department of Radiotherapy Oncology, The Second Affiliated Hospital of Dalian Medical University, Dalian, China

**Keywords:** cervical cancer, HPV oncoproteins, PD-L1/PD-1 pathway, regulators, immunotherapy

## Abstract

Cervical cancer (CC) is a common malignant tumour of the female reproductive system that is highly harmful to women’s health. The efficacy of traditional surgery, radiotherapy and chemotherapy is limited, especially for recurrent and metastatic CC. With continuous progress in diagnostic and treatment technology, immunotherapy has become a new approach for treating CC and has become a new therapy for recurrent and metastatic CC. However, immunotherapy is not effective for all patients with CC. Therefore, factors related to immunotherapy efficacy in CC patients have become the focus of researchers. High-risk human papillomavirus (HPV) infection is an important factor that drives CC development and affects its progression and prognosis. Increasing attention has been given to the mechanism of the E5, E6 and E7 proteins, which are encoded by the HPV gene, in the occurrence and development of CC and their interaction with programmed cell death ligand-1/programmed cell death-1 (PD-L1/PD-1). Although some preliminary studies have been conducted on these topics, a comprehensive and systematic review of these topics is not available. This review comprehensively summarizes related articles from journals with impact factors greater than 3 and published in the past 5 years; it also reviews studies on the mechanism of HPV and CC, the mechanism of PD-L1/PD-1 axis regulation in CC, and the mechanism by which the interaction between HPV-related oncoproteins and the PD-L1/PD-1 pathway affects the development and prognosis of CC. This study provides theoretical support for the use of immunotherapies for CC, provides a basis for the selection of specific medications that target different HPV-related proteins, and provides a new perspective for the discovery of new immunotherapy targets for CC.

## 1 Introduction

Cervical cancer (CC) is one of the most dangerous female cancers worldwide, with more than 604 000 cases of CC reported in 2020 (1, 2). Although various screening methods for CC and the availability of human papillomavirus (HPV) vaccines have made this type of cancer largely preventable (2, 3), the incidence of CC has not decreased significantly (4). HPV is thought to be a cause of CC (5). Previous studies have reported that persistent HPV infection might increase the persistence of cervical intraepithelial neoplasia (CIN), subsequently leading to invasive CC (6–8). HPVs are divided into high-risk and low-risk types according to their level of carcinogenicity (8). Many studies have evaluated the correlation between HPV subtypes and CC progression (9–11) and the prevalent species causing the development of CC. The genes encoding HPVs produce proteins that promote viral DNA replication, cell cycle control, and tumorigenesis in the initial stages of infection (12). The E5 and E6/E7 oncogenes have the most pronounced transformation characteristics (13). Moreover, E6 and E7 are the main regulators of virus pathogenicity (14) and exert carcinogenic effects (15). E6 inhibits cell apoptosis by binding to and marking the p53 protein for degradation. This process results in an inability to repair damaged DNA in infected cells, promoting cell proliferation. E7 binds to the retinoblastoma (Rb) protein and inhibits its function, thereby releasing the inhibition of the cell cycle, allowing cells to enter S phase, and promoting cell proliferation (14, 16, 17). Failure to clear HPV infection in a timely manner can lead to the development of CC (18). Various immunotherapies against CC have been tested for their ability to eliminate viral infections and enhance the immune response. Immunotherapy targeting the programmed cell death ligand-1/programmed cell death-1 (PD-L1/PD-1) axis offers new ideas for treating a wide range of advanced human cancers (19).

**Figure 1:**
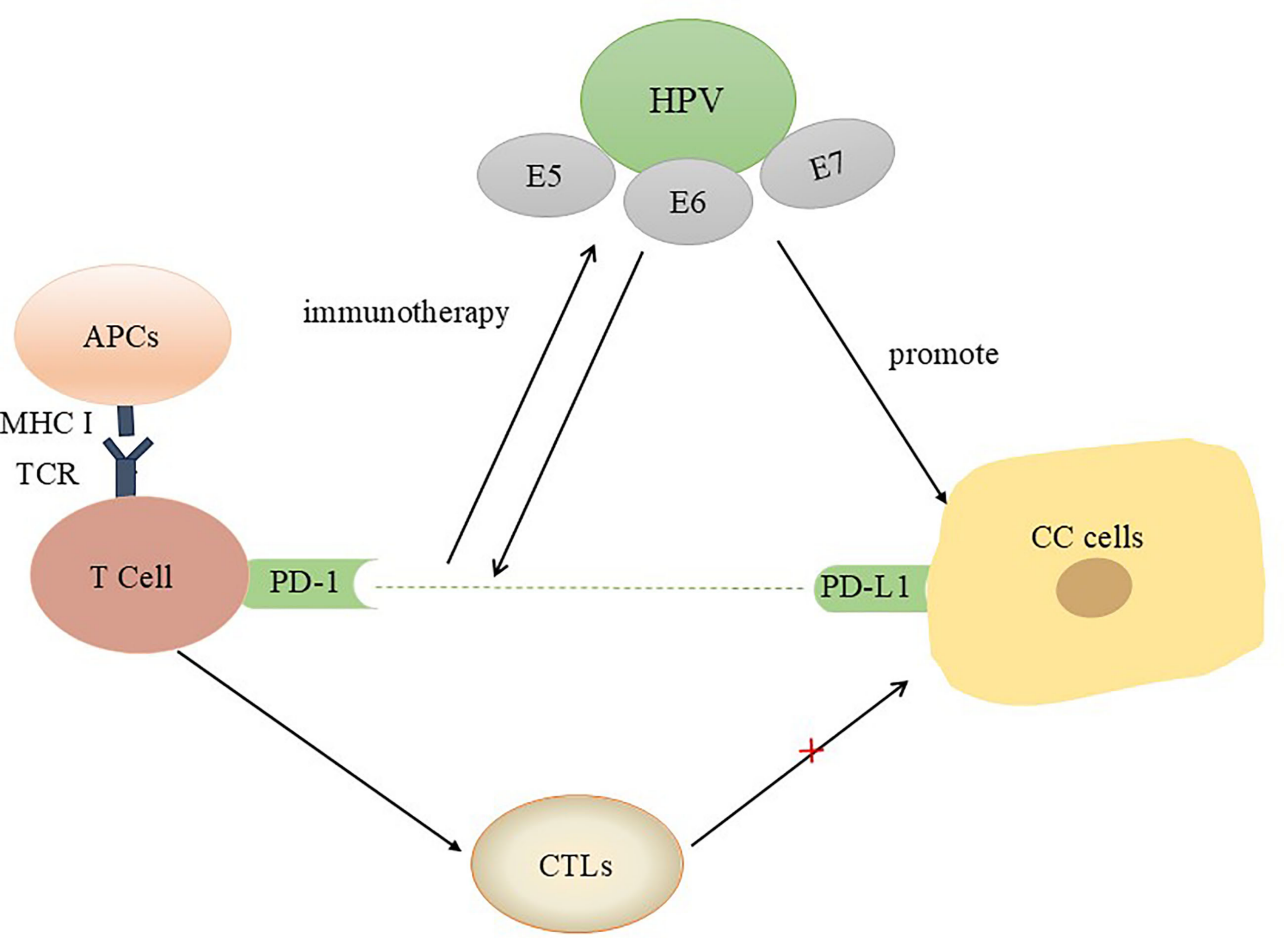
Interactions between HPV oncoproteins and PD-L1/PD-1 in CC patients. In the tumour microenvironment, the interaction between PD-1 and PD-L1 results in the secretion of inhibitory signals and induces T-cell apoptosis. Blocking this pathway is beneficial for activating T cells, reshaping the tumour microenvironment, and preventing immune escape.

In contrast to traditional tumour therapies that directly kill tumour cells, immunotherapy activates the immune system to kill tumour cells by overcoming the immunosuppression caused by tumours and the tumour microenvironment (TME). The tumour microenvironment provides abundant tumour antigens for antigen-presenting cells (APCs) to bind, which promotes their maturation (20). Activated APCs stimulate CD8+ T cells to respond to the presented tumour antigens (20, 21). When CD8+ T cells are activated, they discover and kill tumour cells, which are subsequently converted into cytotoxic T lymphocytes (CTLs) (22, 23). Notably, when a recognizable antigen expressing the major histocompatibility complex (MHC) is present, T cells are activated, and cytokine-producing cells are recruited, which triggers the inflammatory response (24). Activation of the PD-1/PD-L1 pathway is a key cause of HPV-associated CC immune escape (25). By interacting with PD-L1, PD-1 activates signalling pathways that inhibit efferent T-cell activity and act as intermediaries that facilitate tumour cell escape from T-cell killing and thus facilitate tumour cell survival (26, 27). PD-1/PD-L1 inhibitors have proven to be major new advances in anticancer drug treatment, and multiple drugs that block the PD-L1/PD-1 axis have been used to treat 13 types of cancer (28). For example, pembrolizumab has been shown to prevent cancer cells from inhibiting T-cell activation and is the only anti-PD-1 drug approved in the US as a second-line treatment for recurrent CC (29). In addition, a single-group phase 2 trial revealed antitumour activity in patients with PD-L1-positive tumours (combined positive score ≥1) but not in patients with PD-L1-negative tumours (30). Moreover, multiple clinical trials are exploring combination treatment strategies involving immunotherapy (30, 31). Studies have shown that when HPV infection worsens and leads to cervical cytopathy, PD-L1 expression is increased (32), suggesting that HPV infection may suppress the body’s immunity and increase resistance to immunotherapy (**Figure 1**).

This paper reviews a large body of literature and discusses the expression of HPV-related oncoproteins and the important role of HPV in preventing CC progression through PD-L1/PD-1 immunotherapy. This review aims to provide additional insights for future applications of immunotherapy for CC.

## 2 Signalling pathways associated with major HPV oncoproteins

With continuous in-depth research on the molecular mechanism of HPV carcinogenesis, the strong correlation between the activation of HPV oncogenes and cervical lesions has been further revealed. Among them, the most widely studied HPV oncoproteins are the E5, E6 and E7 oncoproteins.

### 2.1 E5 protein

Notably, we summarized many new ways in which CC is affected by HPV oncoproteins, mainly E5, E6 and E7. Kim et al. studied the role of E5 in the progression of CC and reported that the HPV16 E5 oncoprotein promotes CC by upregulating cyclooxygenase-2 (COX-2) expression via the EGFR signalling pathway, in which NF-κB and AP-1 play key roles (33). Moreover, E5 induces EGFR signalling, thus increasing the expression of vascular endothelial growth factor (VEGF), which plays an important role in cancer progression (34, 35). The increase in VEGF expression induced by E5 is also mediated by the activation of MEK-ERK1/2 and PI3K/Akt downstream of EGFR (33).

### 2.2 E6 protein

HPV E6-mediated activation of c-Jun N-terminal kinase (JNK) drives EGFR signalling to promote CC cell proliferation. In this pathway, JNK activated by HPV E6 subsequently phosphorylates and activates c-Jun, which in turn induces the expression of host cell regulatory genes, including EGFR. The pathway is activated when the EGFR-driven signal, in turn, activates JNK. As a result, a complex positive feedback loop is formed in HPV-positive CC cells (36). Furthermore, Shu et al. analysed another regulatory pathway for HPV16 E6, which inhibits p53 transcription by recruiting the coinhibitory factor NCOR1 to activate octamer-binding transcription factor 4 (OCT4) expression (37). Additionally, E6 can promote the degradation of p53 by interacting with the cellular ubiquitin ligase E6AP. This pathway has a neoplastic effect, resulting in escape from cell death (38, 39).

### 2.3 E6 and E7 proteins

Another study analysed another regulatory pathway of p53 through WB assays to detect the expression of p53. Hypoxia-inducible factor-1 alpha antisense RNA-2 (HIF1A-AS2) was found to significantly induce CC cell apoptosis via the P53/caspase9/caspase3 axis mediated by HPV16 E6/E7 (40). Surprisingly, many other possible therapeutic targets are also regulated by the high-risk HPV oncoproteins E6/E7. For example, in knockdown and overexpressing cell culture models, the role of T-box transcription factor 3 (TBX3) in promoting the proliferation and migration of HPV-positive cells was validated, and the tumour-promoting activity of TBX3 in CC was shown to be influenced by HPV E6 and E7 signalling (41). In addition, using RNA-Seq data, Trujillo-Cirilo et al. confirmed that IL-2 receptor (IL-2R) expression was significantly higher in CC tumours than in normal tissues. The HPV E6 and E7 genes increase the activity of the functional IL-2R on CC cells, promoting the onset of CC (42).

Many microRNAs (miRs), which are noncoding regulatory RNA molecules, interact with high-risk HPV oncoproteins (43, 44). For example, miR-34a may be upregulated in E6/E7-expressing cells to promote the development of CC (45). Additionally, E6/E7 may increase the expression of miR-18a to induce SKT4 expression (46). In contrast, another miR, miR-424, is underexpressed in CC tissues and high-grade cervical intraepithelial neoplasia (47, 48). Interestingly, Hong S et al. reported that miR-424 levels were reduced by a factor of 10 in cells expressing E6 and E7. In addition, they confirmed that high expression of miR-424 led to decreased checkpoint kinase 1 (CHK1) levels. A previous study highlighted the important role of CHK1 activation in the genome using general inhibitors (49). Therefore, HPV E6/E7 indirectly leads to high CHK1 expression by inhibiting miR-424, thus affecting viral replication (50). Furthermore, Olmedo-Nieva, L et al. first reported that E6/E7 inhibited RHO 2 family-interacting cell polarization regulator (RIPOR2) expression and increased PFKFB4 expression. These findings, supported by multiple experimental methods, demonstrated that the E6 oncoprotein inhibits RIPOR2 transcription and promotes its ubiquitination, leading to RIPOR2 downregulation (51). Moreover, in a Mexican cohort, RIPOR2 expression decreased with the progression of precancerous cervical lesions, suggesting that downregulated RIPOR2 expression was closely associated with shorter overall survival (OS) (51).

### 2.4 E7 protein

Another study revealed that pyruvate kinase M2 (PKM2) is regulated by the E7 oncoprotein to affect the occurrence of CC (52). HPV16 E7 increases the expression of PKM2 and enhances its nonglycolytic function to promote CC growth, which was confirmed by data from The Cancer Genome Atlas (TCGA) and cell models (52).

**Figure 2:**
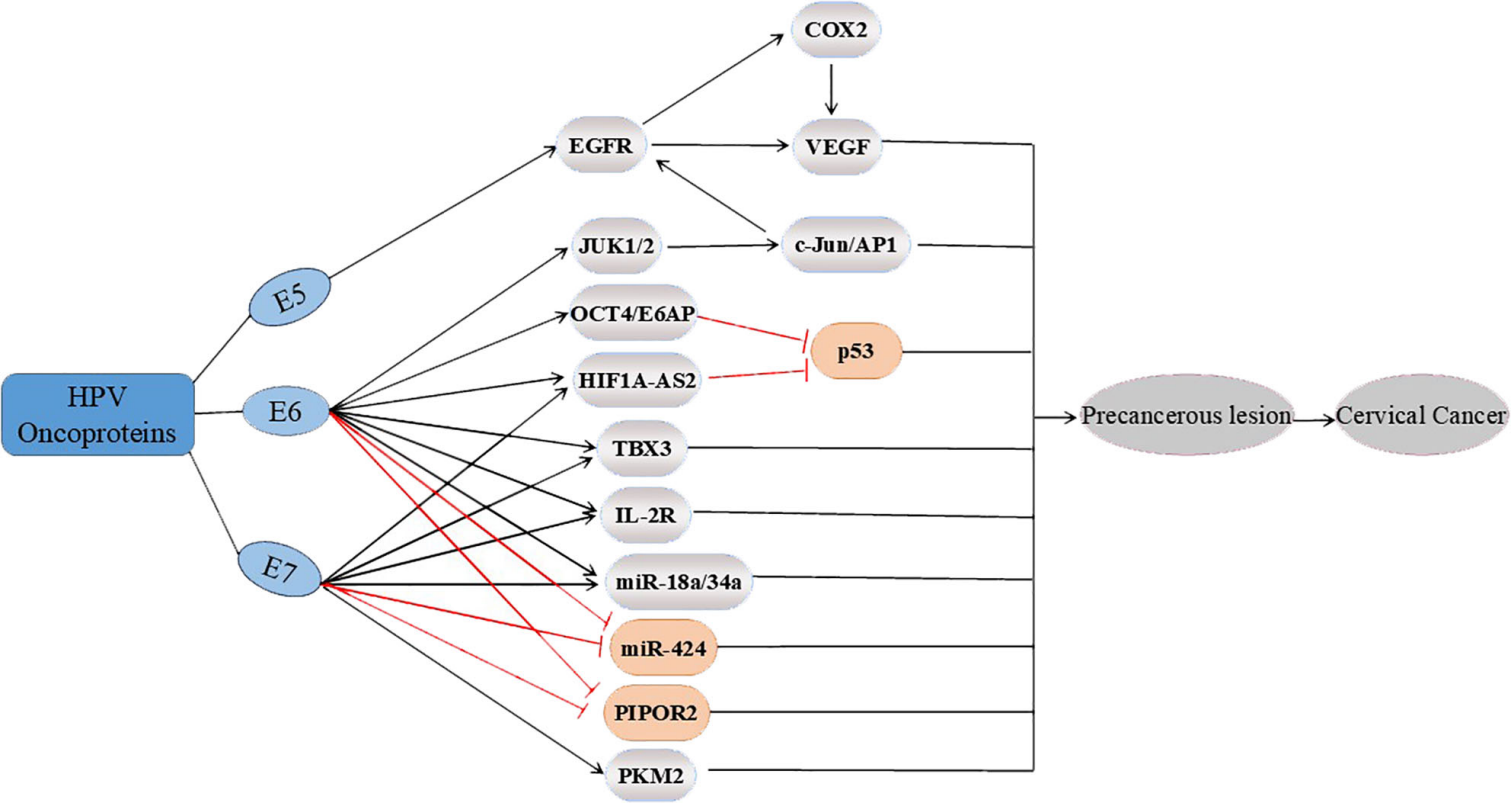
Three major oncoproteins (E5, E6 and E7) affect multiple potential targets involved in the progression of cervical cancer. E5 upregulates EGFR, thereby regulating the expression of COX2 and VEGF; E6 also affects the EGFR pathway by upregulating the expression of JUK1/2 and c-Jun/AP1. In addition, E6 upregulates OCT4/E6AP, and E6 and E7 upregulate HIF1A-AS2, TBX3, IL-2R and miR-18a/34a. OCT4/E6AP and HIF1A-AS2 both affect p53. In addition, E6 and E7 inhibit PIPOR2 and miR-424, whereas E7 upregulates the expression of PKM2, ultimately regulating the progression of cervical cancer.

With the discovery of an increasing number of targets, the role of HPV oncogenic proteins in CC at the molecular level has been confirmed. This finding also reemphasizes the decisive causative role of HPV in CC. The oncogenic proteins E6 and E7 of HPV could influence the process of malignant progression via several common cancer pathways, as shown in **Figure 2**.

## 3 Factors affecting the PD-1/PD-L1 axis in CC

In recent years, breakthroughs in immunotherapy have been achieved in patients with a variety of malignant tumours. Immunotherapy has been used to activate the immune response, which is conducive to the elimination of HPV infection and the early prevention of cancer (53–56). PD-1 and its ligand PD-L1 are immune checkpoints that inhibit tumour-induced immunity (27, 57). PD-1 is found on the surface of diverse immune cells (58). PD-L1 interacts with PD-1 on T lymphocytes and transmits an inhibitory signal (59–61). The inhibition of PD-L1/PD-1 can effectively rescue T cells and improve the prognosis of patients with tumours.

A direct correlation has been reported between elevated PD-L1 expression and shorter overall survival of patients with CC (62). In addition, CC patients who have poor survival outcomes express the PD-L1 protein at high levels; specifically, approximately 96% of tumour samples express PD-L1 (63). In addition, compared with that of PD-1, the expression of PD-L1 can increase in a wider range of cell types in response to inflammatory cytokines and other stimuli. In the TME, PD-L1 expression is regulated by a variety of factors, including inflammatory stimuli and carcinogenic pathways, at the transcriptional, posttranscriptional, and posttranslational levels (64). The therapeutic effects of current immune checkpoint inhibitors can be further enhanced by modulating PD-L1 expression. Exploring the factors affecting the expression of PD-1 and PD-L1 could provide broader ideas for immunotherapy (**Table 1**).

**Table 1:** Different types of regulators of the PD-L1/PD-1 axis.

Target	Regulators	Upstream signals	Effects	References
PD-1	SPOP	CXCL16	SPOP mediates PD-1-induced immune tolerance. Overexpression of SPOP promotes the invasion and metastasis of CC cells *in vitro* and *in vivo*.	(26, 65–67)
	GSK-3	Tbet	Loss of GSK-3 inhibits tumour growth by downregulating PD-1 expression.	(68–70)
PD-L1	miR-21	STAT1	MiR-21 depletion significantly upregulates the expression of PD-L1.	(71, 72)
	IL-1β	NF-κB	IL-1β promotes an increase in PD-L1 expression by activating NF-κB.	(73)
	YY1	p53	High expression of YY1 promotes PD-L1 expression through a variety of signalling pathways.	(74)
	DENR	JAK-STAT	Overexpression of DENR regulates PD-L1 through the JAK-STAT signalling pathway.	(75)
	TAZ	–	TAZ activation *in vivo* promotes tumorigenesis and increases PD-L1 expression.	(76–78)
	FAT4	β-catenin/STT3	FAT4 is responsible for the maladjustment and downregulation of PD-L1 in CC.	(79–81)
	TFAP2A	PI3K-AKT	TFAP2A positively regulates PD-L1 levels in CC.	(82–84)

### 3.1 SPOP

Nuclear spotted poxvirus and zinc finger protein (SPOP) affects the development of multiple malignancies, including but not limited to lung, colon, stomach, and prostate cancers (65). The downregulation of SPOP helps prevent the metastasis of CC and is beneficial for improving the prognosis of CC patients, as shown through *in vivo* and *in vitro* experiments (26). SPOP plays a dual role in CC. SPOP mutations may lead to a decreased ability to degrade the HPV E6 and E7 oncoproteins, thereby increasing genomic stability and allowing cancer cells to survive (66, 67). Additionally, m-IF and HALO analyses revealed that aberrant expression of SPOP reduces the expression of PD-L1 on the cell surface, promoting spatial separation between PD-1 and PD-L1 and thereby promoting immune suppression in the tumour microenvironment (26). CXCL16-related rescue experiments verified that CXCL16 is an intermediate by which SPOP mediates PD-1 immune tolerance (26).

### 3.2 GSK-3

Glycogen synthase kinase 3 (GSK-3, isoforms α and β) is a serine-threonine kinase associated with tumour growth, cell invasion and metastasis (68). Surprisingly, the PI3K-AKT pathway is involved in the regulatory process of GSK-3, further leading to its inactivation (69). Importantly, Taylor et al. reported that inhibition of the enzyme GSK-3 activates the transcription factor Tbx21 (Tbet), which is responsible for the downregulation of PD-1 (70). Therefore, GSK-3 inhibitors may be widely used to modulate immune responses because of their powerful regulatory effect on PD-1. These findings suggest that GSK-3α/β is a regulatory factor involved in PD-1 transcription.

### 3.3 MicroRNA-21

MicroRNA-21 (miR-21) inhibits the STAT1 signalling pathway required for the IFN-γ-induced M1 polarization of macrophages by targeting JAK2 and STAT1, which also regulate apoptosis and carcinogenic transformation (71). Therefore, miR-21 depletion enhances the efficacy of PD-1 antibody immunotherapy through the M1 polarization of TAMs (71). In addition, STAT1 signalling has been shown to transcriptionally modulate PD-L1 expression via IFN-γ in head and neck cancers (72). JAK2/STAT1 signalling is a major coregulator of PD-L1 transcription that is driven by the IFN-γ and EGFR pathways (72).

### 3.4 IL-1β

Xu et al. reported that lactic acid released by tumours can trigger the secretion of IL-1β from Mφs by activating the NLRP3 inflammasome; in turn, Mφ-derived IL-1β suppresses the immune response by activating the NF-κB signalling pathway to drive an increase in PD-L1 levels (73). IL-1β can reduce PD-L1 levels in tumour cells and suppress the infiltration of Mφs *in vivo*, increasing the antitumour efficacy (73).

### 3.5 Yin-yang 1

Overexpression of the transcription factor Yin-yang 1 (YY1) is directly or indirectly involved in the regulation of PD-L1 expression. Many signalling pathways, such as the p53 pathway, are involved in the regulation of YY1 and PD-L1 expression (74). Therefore, targeting YY1 directly or through various pathways engaged in crosstalk may lead to the downregulation of PD-L1 expression in tumour cells, thereby enhancing the cell-mediated antitumour response (74).

### 3.6 DENR

The expression of PD-L1 can be regulated by targeting DENR, which is an RNA-binding protein (RBP) that affects RNA metabolism. Control experiments have shown that in cancer cells lacking DENR expression, the translation of JAK2 and the interferon-γ-JAK-STAT signalling pathway are disrupted, and the level of PD-L1 expression is decreased (75). In summary, DENR is a factor regulating PD-L1 that promotes tumour immune escape. The possibility of DENR as a potential target is an important discovery in immunotherapy.

### 3.7 TAZ

The Hippo pathway interferes with immune cell function and promotes tumorigenesis (76, 77). Transcriptional regulatory factor 1 (TAZ) plays a role in the Hippo pathway. TAZ mRNA expression was significantly increased in the CC group compared with the control group. In addition, a high level of TAZ in CC increases the activity of the PD-L1 promoter (78). The PD-L1 mRNA level in HeLa cells transfected with the TAZ gene was also significantly increased (78). A statistically significant positive correlation was detected between TAZ and PD-L1 protein expression in CC tissues. TAZ targeting of PD-L1 promotes the proliferation and invasion of cancer cells, and TAZ is a major regulator of PD-L1 expression.

### 3.8 FAT4

FAT atypical cadherin 4 (FAT4) functions as a tumour suppressor and has been detected on the cell membrane of mammalian cells (79). FAT4 is abnormally expressed in many tumours due to mutation or deletion, especially in squamous cell carcinoma. The antagonistic effect of FAT4 on the nuclear localization of β-catenin inhibits the β-catenin/STT3/PD-L1 signalling pathway, which is necessary for activating antitumour immunity. In addition, FAT4 induces CTL activation after downregulating PD-L1 expression (80). These findings increase our understanding of PD-L1-related regulation in tumours at the molecular level (81).

### 3.9 TFAP2A

The transcription factor AP−2 alpha (TFAP2A) plays a role in promoting apoptosis in CC. *In vitro* and *in vivo* experiments confirmed that the expression of the TFAP2A gene in CC is higher than that in normal people and is related to the tumour stage and local metastasis. IHC staining was performed for 30 normal cervical tissues and 91 CC tissues, and a higher percentage of PD-L1-positive cells was observed in cancer tissues than in normal tissues (82). The binding of TFAP2A to the PD-L1 promoter region is beneficial for its high expression, thereby forming a positive feedback loop during tumour growth. In colon cancer, the PI3K‒AKT pathway has been shown to be an intermediary for positive feedback (83). However, TFAP2A is also a tumour suppressor; for example, it is underexpressed in hepatocellular carcinoma (84). Therefore, additional studies on the dual role of TFAP2A need to be performed.

Due to the complexity of CC progression and the highly challenging nature of the immunotherapy field (85), the discovery of new therapeutic targets may improve the response rate to immunotherapy. Research on the expression of the PD-1/PD-L1 axis in severe viral infections and HPV-induced CC will be a hot topic.

## 4 Potential roles of HPV oncoproteins in the response to PD-L1/PD-1 immunotherapy

**Figure 3:**
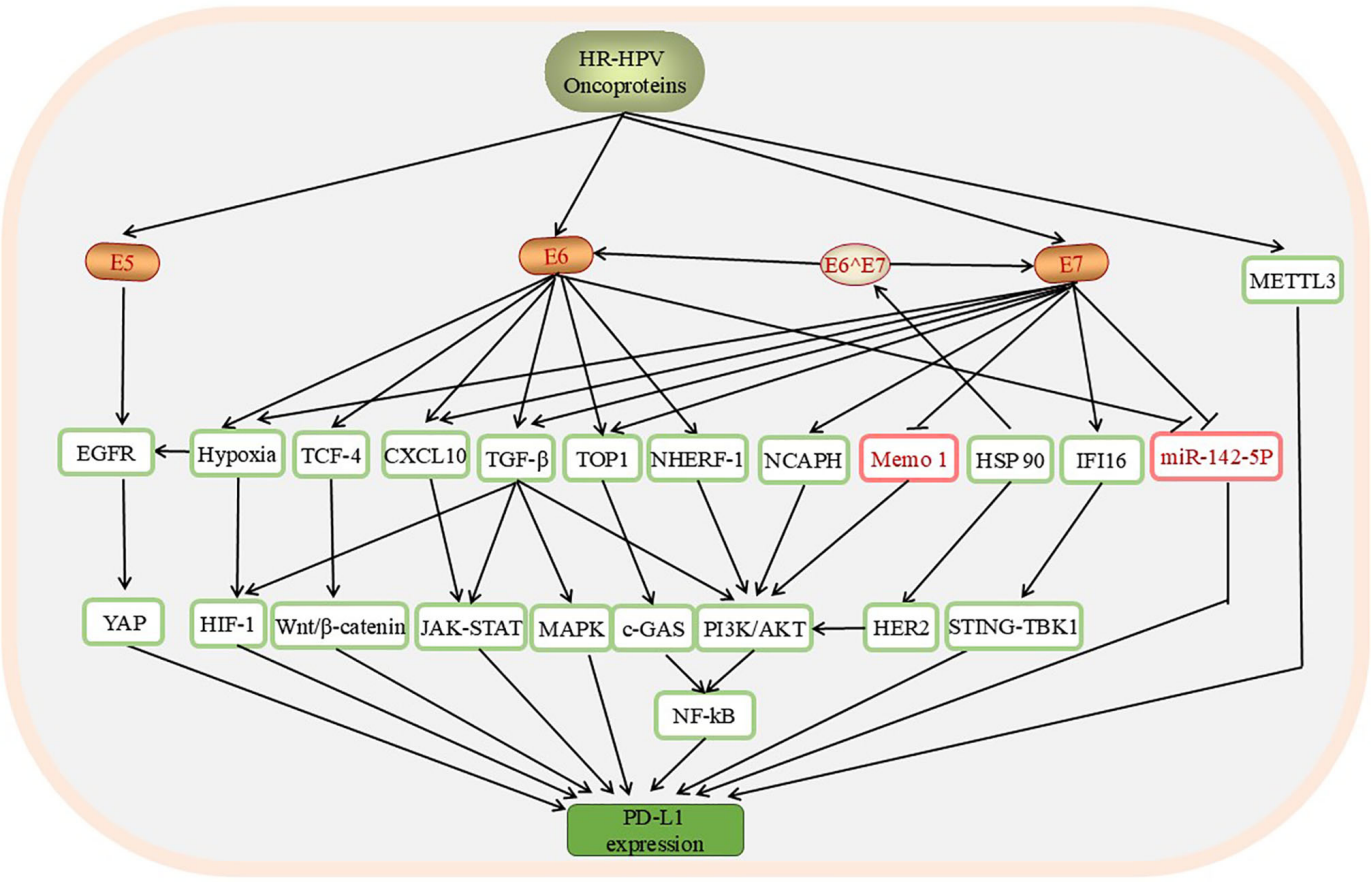
The pathways through which HPV affects the levels of PD-L1/PD-1 in CC include the YAP, HIF-1, Wnt/β-catenin, JAK-STAT, MAPK, c-GAS, PI3K/AKT, and STING-TBK1 pathways. These pathways are involved mainly in promoting PD-L1 mRNA expression. HPV oncoproteins are positive regulators of these downstream factors, except Memo-1, whose expression is downregulated by E7. E6 and E7 inhibit the expression of miR-142-5p, upregulate the expression of PD-L1, and induce the expression of PD-L1. In addition, the upregulation of METTL3 by HPV can increase the expression of PD-L1.

Research on the importance of the PD-L1/PD-1 axis in the aetiology of CC has become increasingly extensive. A study of 25 patients with cervical lesions revealed higher PD-L1 expression in HPV-positive CC patients than in HPV-negative CC patients according to an analysis of a tissue microarray of tumour cores from 25 patients with cervical lesions (*P*<0.05) (86). In addition, the significant correlation between HPV positivity and high PD-L1 expression has been widely studied (32, 87, 88). HPV oncoproteins affect the expression of PD-L1 through a variety of molecular signals (**Figure 3**).

### 4.1 The E5 oncoprotein

Lee et al. reported that yes-associated protein (YAP) affects the activation of the PD-L1/PD-1 pathway by regulating the transcription of PD-L1 (89). The E5 oncoprotein activates EGFR, which in turn enhances the *in vivo* regulation of YAP, thereby increasing the therapeutic potential of YAP and illuminating the complex impact of the EGFR–YAP signalling pathway on PD-L1 in CC progression. In summary, therapy targeting this pathway may downregulate PD-L1 expression and may be a potential mechanism for inhibiting T-cell apoptosis and persistent HPV infection (85, 89–92). Interestingly, Ping-Chih Hsu et al. reported that in NSCLC, PD-L1 expression is also regulated by the EGFR pathway. Activating the EGFR kinase domain is beneficial for further inducing PD-L1 expression, possibly through the activation of the Hippo/YAP signalling pathway, which causes T-cell apoptosis (93). These studies could guide studies of the E5 oncoprotein.

### 4.2 E6 and E7 oncoproteins

The pathways that affect the expression of the PD-L1/PD-1 axis through the E6 and E7 oncoproteins have been studied more extensively.

#### 4.2.1 The HIF-1α pathway

Hypoxia, a physiological stimulus, induces hypoxia inducible factor 2α (HIF-2α) to upregulate the EGFR protein (94, 95). In almost all cancers, the presence of a hypoxic environment increases HIF-1α expression (96, 97). In hypoxic HPV-transformed cells, the expression of the E6 and E7 oncoproteins is downregulated at the transcriptional level via the selective regulation of the PI3K/mTORC2/Akt axis (98–100). More importantly, the E6 and E7 oncogenes increase HIF-1α levels and stability, respectively, in anoxic environments (101). Interestingly, more than one study illustrated that hypoxia increases the expression of PD-L1, and a positive correlation has been observed between HIF-1α and PD-L1 expression in tumours (90, 102, 103).

Under hypoxic conditions, transforming growth factor β (TGF-β) also participates in the HIF-1α signalling pathway and increases the level of PD-L1 in HPV-driven cancers (104). TGF-β can indirectly promote the transcription and upregulation of PD-L1 by activating various non-SMAD-dependent signalling pathways, such as the PI3K/Akt, MAPK/ERK, and JAK/STAT pathways (105). Similarly, in late-stage tumours, the increase in the levels of the HPV E6 and E7 oncoproteins can also activate TGF-β signalling in CC, leading to immunosuppression and tumour progression (106). TGF-β has a dual role. In the early stages of tumour development, E6 and E7 enhancement interferes with the growth inhibition of transformed cells by TGF-β (107). Ultimately, an immunosuppressive microenvironment is established that is conducive to tumour cell escape from the supervision of the immune system. Inhibitors targeting the TGF-β pathway are being used in combination with PD-1/PD-L1 inhibitors to achieve the precise application of immunotherapy. Drugs such as the anti-TGF-β/PD-L1 bispecific antibody YM101 (108) and BITP have seen increased clinical application (109). However, whether HIF-1α-mediated upregulation of PD-L1 is associated with a poor prognosis for CC patients has yet to be investigated.

#### 4.2.2 The Wnt/β-catenin pathway

Overactivation of the Wnt signalling pathway in the progression of CC has been shown to occur in previous studies (110–112). Furthermore, the important role of HPV E6 in this activation pathway has been recognized with the help of cellular models (113). T-cell factor 4 (TCF-4) has been shown to exhibit abnormal mutations in various types of cancer (114). Both E6 and E7 promote Wnt/β-catenin expression and increase TCF-mediated transcription (115, 116). Similarly, Munoz-Bello et al. reported that E6 and E6*I upregulate the TCF-4 transcription factor to regulate the activity of the Wnt/β-catenin pathway, promote the proliferation of cancer cells, induce the stabilization of TCF-4, and help maintain the transformation and immortalization of cancer cells (116). After β-catenin enters the nucleus, it binds to TCF-4, activating the transcription of downstream genes, including MYC (117), which is an important step in driving high PD-L1 expression. Furthermore, both Wnt/β-catenin and PD-L1/PD-1 inactivate CD8+ T-cell function by influencing the c-MYC gene during carcinogenesis and tumour formation, enabling tumour cells to evade the immune system (118). Therefore, inhibition of the Wnt signalling pathway in cancer cells can disrupt PD-L1 expression by affecting MYC signalling, thereby enhancing the immune defence against tumours (119). By targeting various components of the TCF-4-Wnt/β-catenin-MYC-PD-L1 pathway, new diagnostic and therapeutic strategies can be developed to enhance the immune defence against tumours.

#### 4.2.3 The JAK-STAT pathway

CXC motif chemokine 10 (CXCL10) is an important mediator of cancer intracellular signalling pathways and cell transport (120). CXCL10 is secreted by CC cells after HPV infection and subsequently binds to CXCR3 in surrounding fibroblasts (121). Along with the activation of the JAK-STAT pathway, the CXCL10-CXCR3 axis increases extracellular PD-L1 levels (86). These mechanisms may promote virus incubation and help HPVs escape the immune response. Moreover, in gene-edited cells, knocking out E6/E7 leads to a significant reduction in CXCL10 expression, indicating the regulatory effect of HPV E6/E7 on CXCL10 expression in human cervical cells (86, 122). Furthermore, another study revealed that the serum CXCL10 concentration is significantly higher in patients than in healthy controls, which is consistent with previous conclusions (123). Therefore, CXCL10 may be a predictive biomarker for diagnosing CC.

#### 4.2.4 The c-GAS pathway

The upregulation of topoisomerase I (TOP1) triggers DNA repair, acting as a key DDR protein (124). In CC, TOP1 promotes an increase in NF-κB expression by regulating cyclic GMP-AMP synthase (c-GAS), which is an important step in increasing PD-L1 expression. Conversely, a low level of TOP1 inhibits tumour cell growth. Therefore, TOP1 and c-GAS together form a signalling pathway that promotes tumour cell growth (125, 126). Interestingly, the HPV oncogenic protein E6 has been shown to regulate c-GAS (127). Luo et al. further highlighted the roles of the oncogenic proteins E6 and E7 in increasing TOP1 expression. The use of specific shRNAs to suppress the expression of the oncogenic proteins HPV E6 and E7 in CC cells led to the inhibition of TOP1 and PD-L1, with E7 resulting in a greater response rate. Additionally, co-IP experiments revealed that E6 and E7 can promote the interaction between cGAS and TOP1, leading to the accumulation of the PD-L1 protein in cervical cancer patients (128). Furthermore, studies have confirmed that the inhibition of TOP1, such as with topotecan (TPT) or camptothecin (CPT), has antitumour effects (124), driving the activation of the cGAS-NF-κB-PD-L1 pathway in cancer therapy.

#### 4.2.5 The PI3K/AKT pathway

In HPV-induced cancers, NF-κB induces an increase in PD-L1/PD-1 expression, and PI3K/AKT signalling is activated by the E6 and E7 genes (129–131). The continuous NF-κB and PI3K/AKT pathways connect HPV oncogenes with the PD-L1/PD-1 pathway. *In vitro*, the association between the loss of Na+/H+ exchanger regulatory factor-1 (NHERF-1) in CC and ERK signalling stimulated by EGFR has been preliminarily verified (132, 133). Moreover, persistent activation of EGFR by NHERF-1 also results in a poor prognosis for CC patients (133). Moreover, NHERF1 is degraded by E6 and E7, which triggers the PI3K/AKT pathway (134). The direct binding of E2F1 to the nonstructural maintenance of chromosomes (SMC) condensin I complex subunit H (NCAPH) gene promoter provides evidence that E7 increases the expression of the NCAPH gene. When the expression of NCAPH decreases, the expression of PDK1 decreases, which indicates that the activation of the AKT pathway by NCAPH may depend on the upregulation of PDK1 expression (135). Furthermore, NCAPH can transmodulate the transcription of E7 (135).

Unlike NCAPH, Trejo-Cerro et al. discovered the effect of HPV16 E7 on Memo1 through proteomic research and reported that the inhibition of Memo1 promoted the growth of HPV-positive cervical cancer cells, which was accompanied by the activation of the Akt pathway (136). Moreover, the effects of heat shock protein 90 (HSP90) overexpression on E6/E7 cells have been widely analysed. Specifically, HSP90 does not interact directly with E6/E7 but rather stabilizes the viral proteins E6/E7 to exert its effects (137). Furthermore, by exploring the expression pattern of HSP90, Zeng, J et al. confirmed the inhibitory effect of HSP90 knockdown on the proliferation of CaSki and SiHa cells (138). Notably, these findings are consistent with those of previous studies in this field (139). Notably, the downregulation of HSP90 inhibits the HER2/PI3K/AKT pathway, thereby effectively inhibiting the proliferation and migration of HPV16+ cancer cells (138).

#### 4.2.6 The STING-TBK1 pathway

Interferon-gamma inducible factor 16 (IFI16) is an essential protein involved in DNA virus infection that stimulates cGAMP expression (140, 141). HPV E7 facilitates the ubiquitin‒proteasome-mediated degradation of IFI16 through the E3 ligase TRIM21, inhibiting pyroptosis during infection (142). Furthermore, Cai, H et al. injected SiHa cells into NOD/SCID mice to establish tumour xenografts and verify the roles of PD-L1 and IFI16 in CC development. Both PD-L1 and IFI16 knockdown significantly suppressed the growth of SiHa-derived tumours *in vivo* (143). The expression and distribution of IFI16 were analysed using different methods, such as western blotting. The cytoplasmic translocation of IFI16 was affected by IFN-γ stimulation, and IFI16 participated in STING pathway activation through this pathway (144). IFI16 activated STING-TBK1-mediated immunoregulation and the downstream NF-κB pathway, which upregulated PD-L1 in the immune microenvironment, thus promoting CC progression (143). Therefore, STING-TBK1 may constitute a new pathway to inhibit the metastasis of CC cells, which is worth exploring.

#### 4.2.7 MicroRNAs regulate the PD-L1/PD-1 axis

Researchers assume that microRNAs are intermediaries that regulate the expression of other genes. In addition to the miRNAs mentioned above, in recent studies, miR-142-5p has been found to act as a tumour suppressor in CC, mediating the regulatory effect of E6/E7 on PD-L1 (145, 146). The inverse relationship between miR-142-5p and PD-L1 expression was confirmed by the transfection of the E6 and E7 oncoproteins (146). In addition, the upregulation of miR-142-5pweakens the effect of E6/E7 on PD-L1 and inhibits the occurrence of tumours. These results were further confirmed via *in vivo* experiments (146). Therefore, using miR-142-5p as a tumour suppressor to increase the efficacy of tumour immunotherapy is feasible.

#### 4.2.8 Possible factors related to PD-L1 expression in CC

Interestingly, another target that directly affects HPV infection and PD-L1 expression was identified. METTL3 plays an immunomodulatory role in HPV-associated cancers by inhibiting the infiltration of immune cells (147). METTL3, a key regulator of N6-adenosine methylation (m6A), is associated with the HPV status and expression in tumours and is correlated with a poor prognosis (147). A high level of METTL3 promotes the formation of an immunosuppressive tumour microenvironment (147, 148). Additionally, high levels of METTL3 and YTHDF1 may lead to a poor prognosis for patients with CC (149, 150). Moreover, METTL3 accelerates the Warburg effect or aerobic glycolysis (149). Further research by Ji H et al. revealed that METTL3 increased HK2 protein expression by increasing the stability of the HK2 mRNA, thus accelerating glycolysis. Notably, the expression of PD-L1 in CC was negatively correlated with the expression of ALKBH5, FTO, METTL3, RBM15B, YTHDF1, YTHDF3, and ZC3H1 (148). This study has several limitations. For example, how HPV infection affects METTL3 expression remains to be further elucidated. Thus, additional possible interactions between HPV oncoproteins and PD-L1 are worth exploring.

Building on previous research, the continuous enrichment of pathways such as the YAP, HIF-1, Wnt/β-catenin, JAK-STAT, PI3K/AKT, and STING-TBK1 pathways provides new perspectives for trends in personalized immunotherapy and holds promise for addressing the issue of low response rates to immunotherapy.

## 5 Conclusions

HPV infection is one of the important factors leading to the occurrence of CC, particularly high-risk HPV types 16/18, which are the most common. Continuous viral infection is a driving factor in tumour progression, and the HPV oncoproteins E6/E7 can evade host immune surveillance in various ways, ultimately leading to cancer. Numerous studies have confirmed that the expression of PD-L1/PD-1 is related to the treatment and prognosis of CC. However, verification of whether the expression of PD-L1/PD-1 is associated with infection with different types of HPV related to CC is urgently needed. Therefore, understanding the many unelucidated pathogenic mechanisms of HPV, the untapped potential of the PD-L1/PD-1 axis, and the impact of high-risk HPV oncoproteins on the PD-L1/PD-1 pathway is crucial for guiding clinical decisions in selecting effective immunotherapy options for patients with CC. For this purpose, this paper summarizes the high-quality literature published in the past 5 years and elaborates in detail how the E5, E6 and E7 oncoproteins affect the expression of PD-L1 through YAP, HIF-1, Wnt/β-catenin, JAK-STAT, PI3K/AKT and STINT-Tbk1 signalling and promote the occurrence of CC. EGFR, hypoxia, TCF-4, CXCL10, NHERF-1, NCAPH, HSP90, IFI16 and other targets involved in these pathways are positively regulated by oncoproteins. In particular, the Memo1 and miR-142-5p regulatory factors are negatively regulated by the E7 oncoprotein. Targeting or knocking out these factors can effectively hinder the progression of CC cells, significantly enhancing the effect of immunotherapy. Moreover, the expression of PD-L1 itself might predict the clinical outcomes of CC patients. Therefore, we believe that infection with different high-risk HPV types leads to differences in the sensitivity of patients with CC to PD-1/PD-L1 immunotherapy, which provides a reference for individualized and specific clinical treatment and a basis for the development of more effective immune checkpoint inhibitors. Elucidating the roles of different regulatory pathways can help evaluate therapeutic effects and facilitate the prediction of whether the use of a specific immunotherapeutic drug will be effective. Moreover, immunotherapies are expected to largely avoid immune-related side effects and overcome resistance mechanisms. Although the current research is limited, HPV-positive patients are undoubtedly more likely to benefit from the precise application of immunotherapy. The identification of an increasing number of pathways has expanded our understanding of HPV-related CC and, more importantly, strongly validated that immunotherapy may have a wider range of antitumour applications in the future.
